# Determining the genome-wide kinship coefficient seems unhelpful in distinguishing consanguineous couples with a high versus low risk for adverse reproductive outcome

**DOI:** 10.1186/s12881-015-0191-0

**Published:** 2015-07-20

**Authors:** W. Kelmemi, M. E. Teeuw, Z. Bochdanovits, S. Ouburg, M. A. Jonker, F. Alkuraya, M. Hashem, H. Kayserili, A. van Haeringen, E. Sheridan, A. Masri, J. M. Cobben, P. Rizzu, P. J. Kostense, C. J. Dommering, L. Henneman, H. Bouhamed-Chaabouni, P. Heutink, L. P. ten Kate, M. C. Cornel

**Affiliations:** Laboratory of Human Genetics, Faculty of Medicine, University of Tunis El Manar, Tunis, Tunisia; Department of Clinical Genetics, VU University Medical Center, Mail BS7, D450, P.O. Box 7057, 1007 MB Amsterdam, The Netherlands; EMGO Institute for Health and Care Research, VU University Medical Center, Mail BS7, D450, P.O. Box 7057, 1007 MB Amsterdam, The Netherlands; Laboratory of Immunogenetics, Medical Microbiology and Infection Control, Research School V-ICI, VU University Medical Center, Amsterdam, The Netherlands; Department of Epidemiology and Biostatistics, VU University Medical Center, Amsterdam, The Netherlands; Department of Genetics, King Faisal Specialist Hospital and Research Center, Riyadh, Saudi Arabia; Medical Genetics Department, Istanbul Medical Faculty, Istanbul University, Istanbul, Turkey; Department of Clinical Genetics, Leiden University Medical Center, Leiden, The Netherlands; Bradford Institute for Health Research, Bradford Royal Infirmary, Bradford, UK; Division of Child Neurology, Department of Pediatrics, University of Jordan, Amman, Jordan; Department of Pediatric Genetics, AMC University Hospital, Amsterdam, The Netherlands; Genome Biology of Neurodegenerative Diseases, German Center for Neurodegenerative Diseases, Tübingen, Germany; Juliana Children’s Hospital, Hague, The Netherlands; Department of Genetics, Wellcome Trust Brenner Building, St James’s University Hospital, Leeds, UK

**Keywords:** Inbreeding coefficient, Relatedness, Consanguinity, Autosomal recessive disorder

## Abstract

**Background:**

Offspring of consanguineous couples are at increased risk of congenital disorders. The risk increases as parents are more closely related. Individuals that have the same degree of relatedness according to their pedigree, show variable genomic kinship coefficients. To investigate whether we can differentiate between couples with high- and low risk for offspring with congenital disorders, we have compared the genomic kinship coefficient of consanguineous parents with a child affected with an autosomal recessive disorder with that of consanguineous parents with only healthy children, corrected for the degree of pedigree relatedness.

**Methods:**

151 consanguineous couples (73 cases and 78 controls) from 10 different ethnic backgrounds were genotyped on the Affymetrix platform and passed quality control checks. After pruning SNPs in linkage disequilibrium, 57,358 SNPs remained. Kinship coefficients were calculated using three different toolsets: PLINK, King and IBDelphi, yielding five different estimates (IBDelphi, PLINK (all), PLINK (by population), King robust (all) and King homo (by population)). We performed a one-sided Mann Whitney test to investigate whether the median relative difference regarding observed and expected kinship coefficients is bigger for cases than for controls. Furthermore, we fitted a mixed effects linear model to correct for a possible population effect.

**Results:**

Although the estimated degrees of genomic relatedness with the different toolsets show substantial variability, correlation measures between the different estimators demonstrated moderate to strong correlations. Controls have higher point estimates for genomic kinship coefficients. The one-sided Mann Whitney test did not show any evidence for a higher median relative difference for cases compared to controls. Neither did the regression analysis exhibit a positive association between case–control status and genomic kinship coefficient.

**Conclusions:**

In this case–control setting, in which we compared consanguineous couples corrected for degree of pedigree relatedness, a higher degree of genomic relatedness was not significantly associated with a higher likelihood of having an affected child. Further translational research should focus on which parts of the genome and which pathogenic mutations couples are sharing. Looking at relatedness coefficients by determining genome-wide SNPs does not seem to be an effective measure for prospective risk assessment in consanguineous parents.

**Electronic supplementary material:**

The online version of this article (doi:10.1186/s12881-015-0191-0) contains supplementary material, which is available to authorized users.

## Background

Consanguineous marriages – unions between relatives up to the fifth degree – occur in many different parts of the world and are the preferred type of marriage within several populations due to cultural and socioeconomic advantages [1]. Consanguinity is associated with an increase in congenital/hereditary disorders in offspring, in particular autosomal recessive (AR) disorders. An estimated excess of 1.7-2.8 % in morbidity and 3.5 % in mortality has been observed among the offspring of first cousins compared to the offspring of non-consanguineous parents [2]. The risk is dependent on the inbreeding coefficient and the presence of a pathogenic mutation passed on by a common ancestor. Given the estimated excess risk of 1.7-2.8 %, it can be concluded that four times those percentages (6.8-11.2 %) of all first-cousin couples are at high risk of 25 % (or more if they are a carrier couple for more than one AR disorder) for each of their children to be affected by the associated disorder [1, 3]. The risk of being a carrier couple rises substantially with evidence of a family history for a given genetic disorder [2], but often no family history is known and yet the risk is increased. This makes personalized risk assessment difficult, and discriminating between high-risk- (25 % or more) and low-risk couples (comparable to the risk for the general population) often impossible.

In order to provide the best possible risk information and genetic counselling for consanguineous couples before pregnancy, health care providers generally aim to estimate the total risk by both calculating the inbreeding coefficient based on the pedigree and by taking a family history. As mentioned earlier, calculating the actual inbreeding coefficient is difficult, due to complex undocumented genealogies and the fact that DNA identical-by-descent (IBD) between consanguineous partners is subject to variation because of stochastic events during meiosis [3].

In theory, an increase in the proportion of DNA IBD sharing will increase the chance of the presence of pathogenic alleles in both parents and consequently the risk of having children affected by autosomal recessive disorders. In the present study, whose methodology was published earlier [3], the following hypothesis was tested: in a case–control setting, consanguineous parents with a child affected by an autosomal recessive disorder and without a family history of AR disorder have more DNA IBD than similarly related consanguineous parents with only healthy children. If the hypothesis can be confirmed, knowing the genomic proportion of DNA IBD may be helpful to assess risk more precisely in consanguineous couples.

## Methods

### Participants

Information on the in- and exclusion of couples and the number of participants is presented in Table 1. The aim was to include 100 consanguineous couples (cases) with one or more children affected by an autosomal recessive disorder and 100 consanguineous couples (controls) with a family relation comparable to the cases and with only healthy children (at least three). Information on the identity of all first-degree- to third-degree family members of both partners of the couples was obtained as far as this was known to the couple. Cases were excluded if another individual in the family affected by the same disorder was known. Cases were not only included when molecular data were available, but also if the nature of the AR disorder was beyond doubt because of clinical or biochemical confirmation (which was the case in 7/73 cases). AR disorders included ranged from rare to extremely rare. Further details on the methods of ascertainment have been described earlier [3]. It was attempted to have equal numbers of case- and control couples of every ethnic background. Moreover, similar distributions of pedigree relatedness among both the case couples and the control couples were aimed for. There were ten cases where we had a case- and control couple from the same family. The 168 couples originated from 10 different populations (Tunisia, Saudi Arabia, Turkey, Jordan, Morocco, Pakistan, Iraq, Iran, Afghanistan, the Netherlands). Approval for the study was obtained from the Medical Ethics Committee of the VU University Medical Center (the Netherlands), le comité de Protection des Personnes de l’hôpital Charles Nicolle (Tunisia), the Research Ethics Committee (REC) at KFSHRC (Saudi-Arabia), the Clinical Trials Ethical Committee at Istanbul Medical Faculty of the Istanbul University (Turkey) and the IRB committee of Jordan University Hospital (Jordan).

**Table 1:** In- and exclusion of couples

Population		Case couples	Case individuals	Control couples	Control individuals
TUN	Considered eligible	50		102	
	Reliable pedigree and suitable sample:	50		52	
	Blood		100		
	Saliva				104
	Genotype				
	Remaining after DNA quality control	50		52	
	Remaining after data quality control	47		49	
SAU	Considered eligible	14		14	
	Reliable pedigree and suitable sample:	13		14	
	Blood				
	Saliva				
	Genotype		26		28
	Remaining after DNA quality control	13		14	
	Remaining after data quality control	11		12	
TUR	Considered eligible	9		12	
	Reliable pedigree and suitable sample:	9		12	
	Blood		4		
	Saliva		14		24
	Genotype				
	Remaining after DNA quality control	7		10	
	Remaining after data quality control	6		10	
MOR	Considered eligible	4		3	
	Reliable pedigree and suitable sample:	3		2	
	Blood				
	Saliva		6		4
	Genotype				
	Remaining after DNA quality control	3		2	
	Remaining after data quality control	3		1	
JOR	Considered eligible	7		7	
	Reliable pedigree and suitable sample:	2		3	
	Blood				
	Saliva		4		6
	Genotype				
	Remaining after DNA quality control	2		3	
	Remaining after data quality control	2		3	
PAK	Considered eligible	16		16	
	Reliable pedigree and suitable sample:	1		1	
	Blood				
	Saliva		2		2
	Genotype				
	Remaining after DNA quality control	1		1	
	Remaining after data quality control	1		1	
NLD	Considered eligible	1		2	
	Reliable pedigree and suitable sample:	1		1	
	Blood				
	Saliva		2		2
	Genotype				
	Remaining after DNA quality control	1		1	
	Remaining after data quality control	1		1	
AFG	Considered eligible	1			
	Reliable pedigree and suitable sample:	1			
	Blood				
	Saliva		2		
	Genotype				
	Remaining after DNA quality control	1			
	Remaining after data quality control	1			
IRQ	Considered eligible	2		2	
	Reliable pedigree and suitable sample:	1		1	
	Blood				
	Saliva		2		2
	Genotype				
	Remaining after DNA quality control	0		1	
	Remaining after data quality control	0		1	
IRN	Considered eligible	1			
	Reliable pedigree and suitable sample:	1			
	Blood		2		
	Saliva				
	Genotype				
	Remaining after DNA quality control	1			
	Remaining after data quality control	1			
TOTAL	Considered eligible	105		158	
	Reliable pedigree and suitable sample	82		86	
	Remaining after DNA quality control	79		84	
	Remaining after data quality control	73		78	

*TUN* Tunisia, *SAU* Saudi Arabia, *TUR* Turkey, *MOR* Morocco, *JOR* Jordan, *PAK* Pakistan, *NLD* the Netherlands, *AFG* Afghanistan, *IRQ* Iraq, *IRN* Iran)

### Sample preparation, genotyping and quality control

For the couples other than the Saudi Arabian couples, DNA was extracted from the individual’s saliva samples according to standard procedures; for 54 case couples, DNA extracted from blood was available. A total of 66 and 70 non-Saudi case- and control couples respectively were genotyped according to manufacturer’s protocol using Affymetrix 6.0 SNP arrays. The Saudi Arabian couples were subjected to genotyping using Affymetrix 250 K arrays (6 individuals) and Affymetrix Axiom arrays (48 individuals), after DNA extraction from whole blood (See Additional file 1: Table S1).

PLINK was used to perform post-genotyping quality control [4]. Individuals with a genotyping rate of <95 % were excluded. All genotype data were merged for overall analysis; Tunisian, Saudi, and Turkish couples were also merged into separate files for further analysis for each population. Duplicated SNPs were removed and only SNPs were included with a genotype call rate of >95 %. Further quality control included removal of SNPs with a minor allele frequency of less than 5 %. After quality control, 73 case couples and 78 control couples genotyped for 143,512 markers were available for the analysis (see Table 1). To obtain a pruned subset of markers with low linkage disequilibrium, the PLINK-indep-pairwise option was used with parameters *50 5 1.5*. (57,358 SNPs remaining). Multidimensional scaling was performed to analyse the population substructuring by using the PLINK MDS plot option. Results were entered in the statistical package R version 3.0.1 (http://www.r-project.org/) and SPSS version 20 for Windows [5]. The MDS plot was inspected for population clustering and case/control matching.

### Analysis of pairwise relatedness

A kinship coefficient based on the pedigree reported for each couple was calculated according to the method as described by Wright [6].

Although pairwise coefficients of relatedness in genomic data can be calculated based on known allele frequencies in a population, these allele frequencies are frequently not known and often calculated from the sample by estimators of relatedness [7]. Lack of homogeneity of the sample or sampling errors can lead to false estimates [7, 8]. Since in some populations, individuals are genetically more similar than in other countries, to estimate the genomic pairwise relatedness of our sample we used three different estimators (PLINK, King, IBDelphi) to account for the population stratification as well as for the inbreeding in our sample. Moreover, we estimated the relationship coefficients from the whole set of samples (overall analysis), as well as from separate sets containing only couples from one population (population subgroup analysis). This latter analysis was performed only for the Tunisian, Saudi and Turkish couples, given the small sample sizes from the other populations.

PLINK uses a method-of-moments approach where the probability of sharing 0, 1 or 2 SNPs IBD is calculated. The total proportion of SNPs IBD is calculated based on the estimated allele frequency of all SNPs and assumes homogeneity [4]. King uses the same approach, and offers two different methods: King homo, which assumes homogeneity of the sample, and King robust, which provides robust relationship inference allowing for heterogeneity of the sample by a *robust* approach that accounts for population stratification [8, 9]. Finally, IBDelphi is an algorithm that analyses raw data of high-density SNP genotypes from a consanguineous couple by looking for homozygous regions of over 0.5 Mb in both genomes that lack SNPs that exclude IBD [10].

In PLINK, pairwise relatedness between partners of each couple was calculated with the *--genome --rel-check* command in PLINK. In King, the pruned subset of SNPs was used to calculate pairwise IBD through the kinship parameter (for the overall analysis) and homo parameter (for the population subgroup analysis). Finally, individual genotype files were entered pairwise in IBDelphi, producing IBD measures. All estimates of pairwise relatedness (pedigree, PLINK, King and IBDelphi) were entered in the statistical package R and SPSS version 20. Pearson’s correlation coefficients were calculated for correlations between the different estimates. Rgen represents the relatedness as derived from the genotype, while Rped was calculated based on the pedigree information reported.

The ratio R = (Rgen-Rped)/Rped was used as a measure of the degree of similarity between Rgen and Rped, with Rgen being the observed measure of pairwise relatedness (resulting from our analyses by the four different approaches) and Rped the kinship coefficient between the parents of a child based on the pedigree. If, for a couple, Rped is higher (lower) than Rgen, R is negative (positive). By dividing the difference by Rped, we consider the relative differences. The possible influence of population was ignored first, and the alternative hypothesis was tested that the median of the distribution of ratio R of cases (couples with affected children) is higher than the median of the distribution of controls (couples with only healthy children) with the one-sided non-parametric Mann Whitney test at level 0.05. Since most (96 of the 151 couples) couples come from Tunisia, they were subsequently selected to filter out a possible population effect, and the same test was performed based on these selected data. The analyses were also done separately for the first cousin couples (based on pedigree) as they are the most predominant consanguineous couples who seek genetic counselling.

Next, a mixed effects linear model was assumed. The outcome variable in the model is equal to ratio R, the covariates consist of an intercept, the fixed effect 0–1 variable “whether the couple has an affected child (covariate equals 1) or not (covariate equals 0)” (i.e. case or control), and a random effect “population”. The population effect on the association between the outcome variable R and the case–control status was investigated and the one-sided alternative hypothesis was tested regarding whether the regression parameter for case–control status was positive, corrected for a possible population effect if “population” is a confounder.

## Results

Seventy-three case- and 78 control couples from 10 different populations were analysed (Table 1; Additional file 1: Table S1). Sixty-five of the 73 case couples had a child with an autosomal recessive disorder that was diagnosed by molecular testing, two case couples had a child that was diagnosed by biochemical testing, and the remaining six case couples had a child with a clinical diagnosis for an AR disorder. The respective AR disorders are presented as Supplementary Information. Control couples had on average 4.2 children (SD = 1.6), ranging from three to 11.

### Substructuring within the different ethnicities

**Fig. 1:**
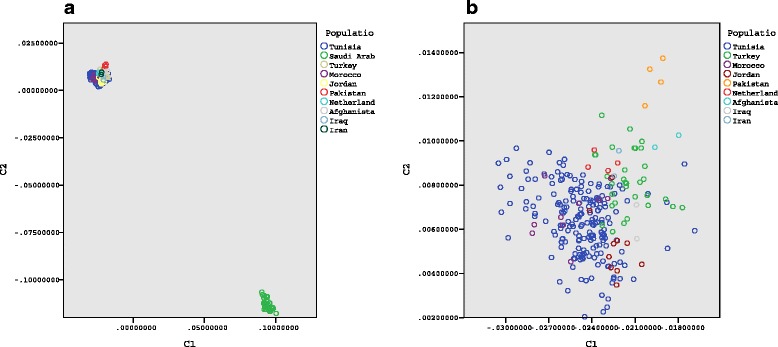
**a** shows an MDS two-dimensional plot of all individuals in the sample, showing separate clustering of Saudi Arabian individuals (lower right corner). **b**: enhancement of upper left corner of Fig. 1a

**Fig. 2:**
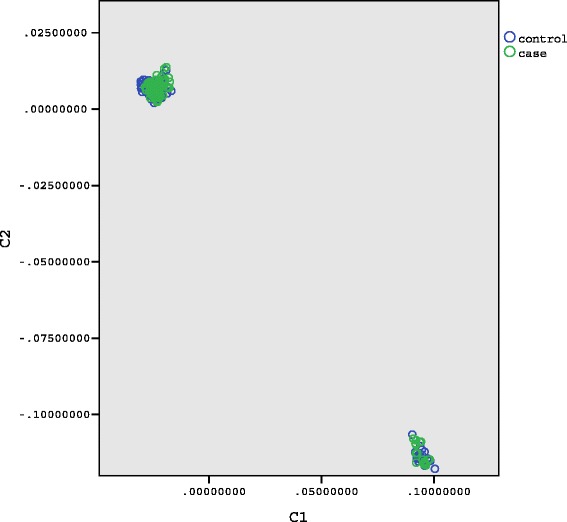
MDS two-dimensional plot of all individuals in the sample. Cases indicated with green dots, controls with blue dots

In Fig. 1 two components resulting from within-group multidimensional scaling analysis of all individuals are plotted against each other in a scatter plot. As expected, evidence of substructuring within the different ethnicities is present, with each population distinguishable from the others. Saudi individuals are observed to cluster separately from the other populations in the lower right corner of the figure as a result of using a different marker set for the genotyping. To explore comparability of cases and controls, they were plotted separately (Fig. 2). This shows consistent clustering by population, with an apparent overlap of cases and controls.

### Relationship inference estimates

The estimated degrees of relatedness show a substantial variability between the different estimators (Table 2). PLINK (all) kinship coefficients estimates resulted in a mean for case couples of 0.110 (SD = 0.06), the mean for control couples was 0.108 (SD = 0.05). King robust (all) estimates for all samples are lowest (cases: mean = 0.039 (SD = 0.035) vs. controls: mean = 0.050 (SD =0.031)). In contrast to our hypothesis, controls show higher point estimates for degree of genomic relatedness. Despite the differences in level of the estimate, correlation measures between the different estimators show moderate to strong correlations. Pearson’s correlation coefficients were greater or equal to *r* = +0.739, *p* < 0.001 (Table 3).

**Table 2:** Mean and standard deviation of pedigree-based relatedness coefficient and relatedness inference estimates for different estimators

	Mean (standard deviation)	
	Case	Control
Pedigree	0.110 (0.058)	0.108 (0.049)
PLINK (all)	0.151 (0.064)	0.164 (0.054)
King robust (all)	0.039 (0.035)	0.050 (0.031)
PLINK (by population)	0.126 (0.063)	0.141 (0.051)
King homo. (by population)	0.054 (0.034)	0.062 (0.030)
IBDelphi	0.121 (0.059)	0.133 (0.053)

all whole sample; by population separate estimates for Tunisian, Saudi and Turkish couples

**Table 3:** Pearson’s correlation coefficient between estimators

	IBDelphi	PLINK (all)	King robust (all)	PLINK (by pop.)	King homo (by pop.)
PLINK (all)	.947	1	.880	.963	.962
King robust (all)	.739	.880	1	.814	.831
PLINK (by pop.)	.926	.963	.814	1	.964
King homo (by pop.)	.919	.962	.831	.964	1
IBDelphi	1	.947	.739	.926	.919

*P*-values were all <0.001. all whole sample; by pop. separate estimates for Tunisian, Saudi and Turkish couples

### Genomic estimates compared to pedigree-based estimates

**Fig. 3:**
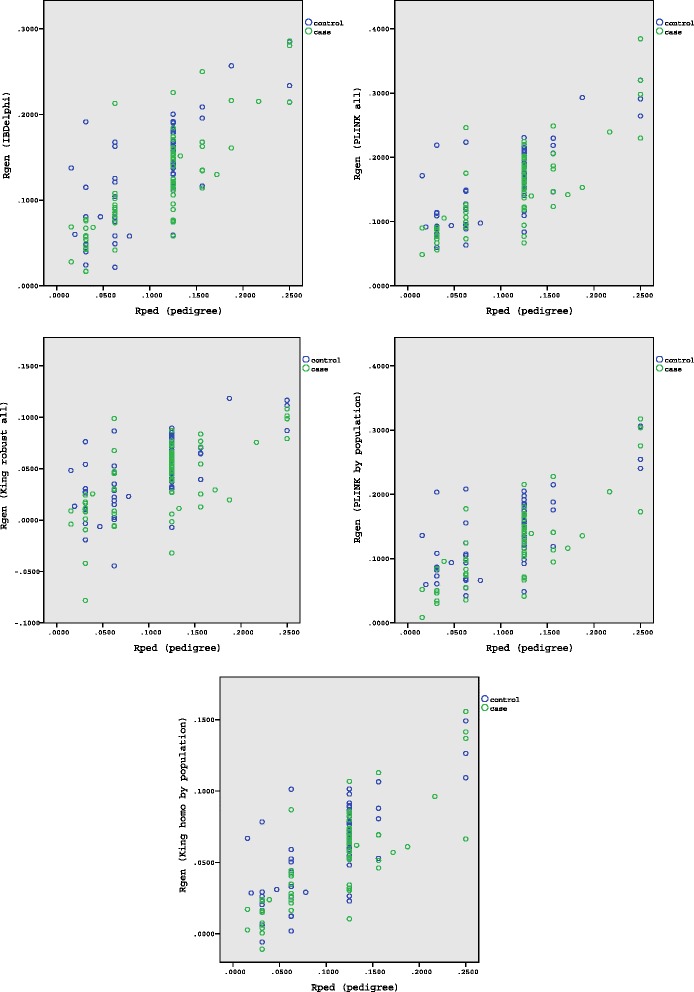
Pedigree kinship coefficient plotted against kinship coefficient estimates of different estimators. Cases indicated with green dots, controls with blue dots.

The results of the genomic estimates of the estimators were plotted against the relatedness coefficient based on the pedigree (Fig. 3). As expected, higher pedigree-based estimates, on average, correlate with higher genomic estimates. In a proportion of couples, genomic relatedness is much increased compared to the expected relatedness based on the pedigree, resulting in a skewed distribution of relatedness towards higher values, which can be explained by hidden consanguineous loops.

### Comparing estimates of IBD in cases to IBD in controls

The PLINK (all) mean and median value of the genetic difference ratio R in the sample of couples with affected children are equal to 0.638 and 0.454, respectively, whereas these values unexpectedly increase to 0.892 and 0.514 in the sample of couples with unaffected children. Our hypothesis was thus not confirmed. The considerable difference between the means and the medians are caused by a few couples with high R-values (i.e. R ≥ 3). This was the case in 4 couples in the PLINK (all) analysis. For the other estimators this involved: 0 couples (King robust (all)), 2 couples (PLINK (by population)), 1 couple (King homo (by population)) and 3 couples (IBDelphi).

For ten case couples, a control couple from the same family was available. The association between their R-values was computed and considered by making scatter plots. Since no association was seen, the R-values of case couples and control couples within a family are assumed to be independent in the further analysis. The one-sided Mann Whitney test based on the whole data set gave a p-value of 0.907. The other three methods for estimating DNA IBD as well as the separate analyses for first cousin couples yielded similar p-values (Table 4). Based on this test, it cannot be concluded that couples with affected children are genetically more similar than expected, based on their kinship relatedness, than couples without affected children. Next, the parameters in the mixed effects linear model were estimated. In all models, “population” seems not to be a confounder and was left out of the model (the estimated standard deviation was very small compared to the estimated standard deviation for the measurement error in the model). Also including the covariate “Rgen” into the linear model gave a better fit, but the association between the variable “case–control status” and the outcome variable hardly changed. This was expected due to the design of the study. It was tested whether the regression parameter for the covariate case–control status was significantly bigger than zero in the linear model. In the latter analysis, several influence points were left out. With PLINK (all) measures, the regression parameter was estimated as −0.028 and the alternative hypothesis that the regression parameter is positive was not rejected. Estimates and p-values for the other methods can be found in Table 4. The conclusion still holds true: it cannot be concluded that couples with affected children have more DNA IBD than couples with only healthy children.

**Table 4:** Results of Mann Whitney test and mixed effects linear model for ratio R

		Mean / Median		Mann Whitney test	Mixed effects model	
		Case	Control	*p*-value	β	*p*-value
PLINK (all)	All couples	0.276 / 0.313	0.347 / 0.338	0.907	−0.028	0.625
	1^st^ cousins^a^	0.290 / 0.320	0.410 / 0.404	0.980		
King robust (all)	All couples	−0.615 / -0.595	−0.462 / -0.534	0.990	−0.152	0.992
	1^st^ cousins^a^	−0.580 / -0.550	−0.520 / -0.510	0.950		
PLINK (by population)	All couples	0.209 / 0.145	0.594 / 0.233	0.991	−0.157	0.965
	1^st^ cousins^a^	0.080 / 0.130	0.200 / 0.180	0.950		
King homo (by population)	All couples	−0.506 / -0.490	−0.341 / -0.428	0.996	−0.086	0.980
	1^st^ cousins^a^	−0.490 / -0.474	−0.430 / -0.428	0.940		
IBDelphi	All couples	0.278 / 0.192	0.479 / 0.252	0.854	−0.085	0.834
	1^st^ cousins^a^	0.058 / 0.057	0.160 / 0.160	0.950		

^a^ Based on pedigree

Another way of accounting for a possible population effect is by selecting individuals from a single population and performing the Mann Whitney test based on this subset only. For the Tunisian data only (96 couples), PLINK (by population), King homo (by population) and IBDelphi, a Mann Whitney test was performed separately. For PLINK (by population), the mean and the median of ratio R equal respectively 0.132 and 0.073 for the couples with an affected child and 0.510 and 0.182 for those without an affected child. The couples without an affected child show, on average, a higher genomic kinship relatedness than expected compared to the couples with an affected child. The p-value of the one-sided Mann Whitney test now equals 0.990. For the results of the other estimators, see Table 5. So, by restricting the analysis to Tunisian couples, it cannot be concluded that couples from Tunisia with an affected child have more DNA IBD than expected based on their kinship relatedness than couples from Tunisia without affected children. The values ratio R mean and median rather show the reverse of what was expected. The three other methods for estimating IBD all lead to the same conclusion.

**Table 5:** Results of Mann Whitney test Tunisian subpopulation for ratio R^a^

	Mean / Median		Mann Whitney test
	Case	Control	*p*-value
PLINK (by population)	0.132 / 0.073	0.510 / 0.182	0.990
King homo (by population)	−0.529 / -0.520	−0.372 / -0.440	0.982
IBDelphi	0.275 / 0.165	0.426 / 0.165	0.815

^a^ Only measures by population are presented given the relative homogeneity of the sample

## Discussion

The hypothesis that consanguineous couples with an affected child have, on average, a higher amount of DNA IBD than consanguineous couples with only healthy children was not confirmed by this study. For clinical purposes determining the amount of DNA IBD can, therefore, not differentiate between consanguineous couples at high risk vs. those at low risk. Results from PLINK and IBDelphi show that both case couples and control couples share on average more DNA IBD than expected based on the pedigree. The same results applied to both first cousins and consanguineous couples all combined. This is a phenomenon more often found especially in populations with a tradition of consanguinity, where the proportion of DNA IBD is larger due to hidden consanguineous loops in the extended family [11]. Moreover, extended tracts of homozygosity have been found more generally in populations not even associated with recent consanguinity [12–15].

In this study we have ascertained cases and controls from a population at increased risk, namely consanguineous couples. The exact magnitude of DNA IBD does not seem to make any difference between the couples who did have an affected child – and were thus revealed as carriers of the same pathogenic mutation – and those who had only healthy children. Chance plays a role in the passing of parental mutations to the next generation, and thus in having two pathogenic mutations in a child. It is possible that our selection of couples contained control couples who, in reality, are carrier couples for the same pathogenic mutation but have had only healthy children. However, the chance of being a couple *without* a similar pathogenic mutation is a priori already high. For first cousins this is approximately 88.8-93.2 % (100 % minus four times the risk for first cousins (1.7 – 2.8 %), for second cousins 97.2-98.4 % as their risk is four times lower than the risk for first cousins [3, 16]. Only couples were selected with three children at least (increasing these chances even more), leading to a small differentiation in a posteriori chances between the different numbers of healthy children. It was therefore decided not to apply weighting according to the number of children.

In order to answer our research question, we performed one-sided tests rather than two-sided tests, since it was hypothesized that case couples have more DNA IBD than control couples. From the values of the means and medians as well as from the estimate of the regression parameter in the (mixed) regression model, we see an opposite effect in our data: the control couples have more DNA IBD than expected based on their kinship coefficient than the couples with an affected child. This could be due to recall bias where control couples may have reported less consanguineous relationships in the family than case couples who were counselled in clinical genetic centres because of their affected child or children. Case couples may have made more effort earlier when confronted with the autosomal recessive disorder of their child to report the family history and family loops, resulting in more awareness regarding consanguinity in their family. This may have led to a relatively lower Rped for controls and thus a higher ratio R. Another explanation could be the fact that control couples originated from more endogamous subpopulations, but this would only serve as a possible explanation in, for example, the Tunisian population, and not in the Saudi population (where most control couples were from the same family). If the trend towards controls sharing at least as much DNA IBD as cases is in fact a real finding, more research is needed to look into possible explanations, like differences in the nature of the shared segments. Genes are unequally distributed throughout the genome [17, 18] and consanguineous couples can as a result inherit more or less ‘quiet’ regions.

This study has several limitations. Although the initial goal was to ascertain an intrafamilial control for every case couple, the lack of available and suitable candidates eventually resulted in only 10 cases for which this was achieved.

In our power calculation we reasoned as follows: we assumed that for first-cousin couples, who theoretically have 0.125 of their genome IBD, a standard deviation of 0.0625 would apply, and we expected to have sufficient power (90 %) with 100 cases and 100 controls, counting a possible loss of 15 %. However, ascertainment was difficult and the sample turned out rather heterogeneous. Eventually, we found 73 versus 78 couples instead of the 85 vs. 85 that we anticipated. The results do not imply that a lack of power in our study was the reason for not being able to confirm our hypothesis. Therefore, including more couples will not likely lead to a considerable effect size and a possible clinical application of estimating DNA IBD in consanguineous couples.

Estimating relatedness coefficients is known to be complicated, especially in the case of population stratification and/or inbreeding [8, 19]. Our sample contains a variety of ethnicities and individuals from populations with a tradition of consanguinity. The heterogeneity of the sample might be causing inflated estimates of kinship coefficients due to population stratification. Because of the heterogeneity of the sample, SNPs with a relative lower frequency in the sample, will be assigned an IBD status sooner than might have happened if the whole sample was from the same population. However, this principle applies to both the case and the control group. Moreover, the three different estimators were consistent in *not* showing that cases share more DNA IBD than controls. The IBD estimation in the overall analysis might also be inflated compared to the actual IBD value because of the relative homogeneity of the Saudi group versus the non-Saudi group. However, both the IBDelphi approach and the separate population approach were not influenced by the differences in the applied marker sets and they show comparable results.

## Conclusion

In this study, consanguineous partners that have a child with an AR condition and most often are both carrier of the same mutation for an AR disorder, do not share more DNA IBD than similarly related consanguineous couples that do not show evidence of carrier status of an AR disorder. In order to assess the risk for a consanguineous couple in clinical practice in the preconception phase, measuring the amount of DNA IBD does not seem to be an effective approach. Further research on other genomic techniques to improve risk assessment for consanguineous couples is needed as current approaches are often unable to identify couples at high risk of having affected children [3]. A promising possibility may be the application of exome sequencing in the preconception phase, although there are still many challenges to face, like dealing with variants of unknown significance or mutations that reside in the uncovered regions by exome sequencing [20, 21]. Whether preconceptional carrier testing for a number of AR disorders, by tailor-made analysis for a panel of disorders for the population from which the couple originates, is a less expensive alternative and thus more feasible, remains to be seen.

## Additional file

Additional file 1:
**A. Specification of disorders in children of case couples,** ***n*** **=73.** B. Specification of control couples (*n*=78).
