# Supplementary material for: Determining the genome-wide kinship coefficient seems unhelpful in distinguishing consanguineous couples with a high versus low risk for adverse reproductive outcome
Source: BMC Med Genet. 2015 Jul 20;16:50. doi: 10.1186/s12881-015-0191-0 (PMC4557855; doi:10.1186/s12881-015-0191-0)
Supplement: Additional file 1: — A. Specification of disorders in children of case couples, n =73. B. Specification of control couples (n=78). [file 12881_2015_191_MOESM1_ESM.pdf]

Table S1A. Specification of disorders in children of case couples, n=73

CASE	POP	Rped	PLINK (all)	King robust (all)	PLINK (by pop.)	King homo (by pop.)	IBDelphi	AR Disorder	DIAGNOSIS
1	TUN	0.0625	0.1207	0.0471	0.0742	0.0283	0.0785	AR deafness 3 (MIM#600316)	Molecular
2	TUN	0.0625	0.1142	0.0301	0.0833	0.0404	0.0856	AR deafness 7 (MIM#600974)	Molecular
3	TUN	0.125	0.1494	0.0523	0.1097	0.0535	0.1115	AR deafness 6 (MIM#600971)	Molecular
4	TUN	0.0625	0.1066	0.0302	0.0552	0.0263	0.083	AR deafness 2 (MIM#600060)	Molecular
5	TUN	0.125	0.1592	0.0625	0.1313	0.0644	0.1118	AR deafness 2 (MIM#600060)	Molecular
6	TUN	0.25	0.23	0.0986	0.173	0.0664	0.2142	Congenital Adrenal Hyperplasia due to 21-Hydroxylase Deficiency (MIM#201910)	Molecular
7	TUN	0.0313	0.0561	0.0116	0.0302	0.0039	0.017	AR deafness 31 (MIM#607084)	Molecular
8	TUN	0.125	0.1212	0.0466	0.0698	0.0304	0.0743	AR deafness 12 (MIM#601386)	Molecular
9	TUN	0.0625	0.2462	0.0989	0.1776	0.0869	0.213	Spinal Muscular Atrophy type I (MIM#253300)	Molecular
10	TUN	0.1563	0.1868	0.0712	0.1405	0.0691	0.1351	AR deafness (MIM#612644)	Molecular
11	TUN	0.125	0.1651	0.0269	0.1448	0.0714	0.1744	Criggler Najjar Type 1 (MIM#218800)	Molecular
12	TUN	0.125	0.202	0.0638	0.1705	0.0842	0.1738	Criggler Najjar Type 1 (MIM#218800)	Molecular
13	TUN	0.1563	0.1233	0.0129	0.0949	0.0461	0.1141	Congenital Adrenal Hyperplasia due to 21-Hydroxylase Deficiency (MIM#201910)	Molecular

14	TUN	0.125	0.1978	0.0505	0.1705	0.0841	0.1842	Congenital Adrenal Hyperplasia due to 21-Hydroxylase Deficiency (MIM#201910)	Molecular
15	TUN	0.125	0.184	0.0621	0.1676	0.0827	0.1613	Familial Mediteraneen Fever (MIM#249100)	Molecular
16	TUN	0.0313		-0.0782		-0.0108	0.0468	Familial Mediteraneen Fever (MIM#249100)	Molecular
17	TUN	0.1563	0.1468	0.0253	0.1134	0.0514	0.1344	Spinal Muscular Atrophy type I (MIM#253300)	Molecular
18	TUN	0.125	0.1888	0.0655	0.1613	0.0796	0.154	Spinal Muscular Atrophy type I (MIM#253300)	Molecular
19	TUN	0.125	0.077	-0.0016	0.0415	0.0105	0.089	Familial Mediteraneen Fever (MIM#249100)	Molecular
20	TUN	0.1563	0.2488	0.0837	0.2278	0.1129	0.25	Familial Mediteraneen Fever (MIM#249100)	Molecular
21	TUN	0.0313	0.0669	0.0082	0.0343	0.0159	0.0167	Familial Mediteraneen Fever (MIM#249100)	Molecular
22	TUN	0.125	0.1368	0.0395	0.1065	0.0522	0.1116	Familial Mediteraneen Fever (MIM#249100)	Molecular
23	TUN	0.0625	0.0732	0.0088	0.0355	0.0164	0.0417	Familial Mediteraneen Fever (MIM#249100)	Molecular
24	TUN	0.125	0.1603	0.044	0.1396	0.0688	0.1527	Familial Mediteraneen Fever (MIM#249100)	Molecular
25	TUN	0.1875	0.1531	0.0197	0.1356	0.061	0.161	Congenital Adrenal Hyperplasia due to 21-Hydroxylase Deficiency (MIM#201910)	Molecular
26	TUN	0.0313	0.087	0.0163	0.0503	0.024	0.054	Congenital Adrenal Hyperplasia due to 21-Hydroxylase Deficiency (MIM#201910)	Molecular
27	TUN	0.125	0.1826	0.0728	0.1403	0.0657	0.1456	Congenital Adrenal Hyperplasia due to 21-Hydroxylase Deficiency (MIM#201910)	Molecular
28	TUN	0.0156	0.0899	0.0089	0.052	0.0171	0.0688	Congenital Adrenal Hyperplasia due to 21-Hydroxylase Deficiency (MIM#201910)	Molecular

29	TUN	0.125	0.0943	0.0058	0.1035	0.0316	0.0768	Congenital Adrenal Hyperplasia due to 21-Hydroxylase Deficiency (MIM#201910)	Molecular
30	TUN	0.0313	0.0731	0.0087		0.0005	0.0775	Congenital Adrenal Hyperplasia due to 21-Hydroxylase Deficiency (MIM#201910)	Molecular
31	TUN	0.125	0.2001	0.077	0.1616	0.0796	0.1552	Congenital Adrenal Hyperplasia due to 21-Hydroxylase Deficiency (MIM#201910)	Molecular
32	TUN	0.125	0.182	0.0743	0.1487	0.0733	0.1501	Bardet Biedel Syndrome8 (MIM#209900)	Molecular
33	TUN	0.1719	0.1419	0.0294	0.1163	0.057	0.13	Bardet Biedel Syndrome5 (MIM#209900)	Molecular
34	TUN	0.1563	0.1818	0.0546	0.141	0.0694	0.1628	Bardet Biedel Syndrome (MIM#209900)	Clinical (molecular ongoing)
35	TUN	0.125	0.1622	0.0599	0.1313	0.0646	0.1146	Bardet Biedel Syndrome12 (MIM#209900)	Molecular
36	TUN	0.125	0.1228	0.0382	0.0717	0.0343	0.0757	Bardet Biedel Syndrome2 (MIM#209900)	Molecular
37	TUN	0.125	0.171	0.0552	0.1505	0.0742	0.1395	Bardet Biedel Syndrome1 (MIM#209900)	Molecular
38	TUN	0.0156	0.0486	-0.004	0.0084	0.0027	0.028	Bardet Biedel Syndrome1 (MIM#209900)	Molecular
39	TUN	0.1328	0.1398	0.0113	0.1391	0.062	0.1517	Bardet Biedel Syndrome7 (MIM#209900)	Molecular
40	TUN	0.125	0.2245	0.0667	0.2155	0.1068	0.2256	Bardet Biedel Syndrome2 (MIM#209900)	Molecular
41	TUN	0.0313	0.0785	0.0081	0.0498	0.015	0.0758	Bardet Biedel Syndrome1 (MIM#209900)	Molecular
42	TUN	0.125	0.1182	0.0276	0.0666	0.0322	0.0956	Bardet Biedel Syndrome8 (MIM#209900)	Molecular
43	TUN	0.125	0.1786	0.0571	0.1492	0.0735	0.1706	Criggler Najjar Type 1 (MIM#218800)	Molecular
44	TUN	0.25	0.2981	0.1081	0.2756	0.1369	0.2859	Criggler Najjar Type 1 (MIM#218800)	Molecular

45	TUN	0.0625	0.0908	-0.0054		0.0237	0.0914	Familial Mediteraneen Fever (MIM#249100)	Molecular
46	TUN	0.0625	0.0994	-0.0064	0.1241	0.0347	0.0893	Congenital Adrenal Hyperplasia due to 21-Hydroxylase Deficiency (MIM#201910)	Molecular
47	TUN	0.125	0.1719	0.0648	0.1265	0.0621	0.1212	Congenital Adrenal Hyperplasia due to 11-Beta-Hydroxylase deficiency (MIM#202010)	Molecular
48	SA	0.125	0.1526	0.0529	0.1149	0.0527	0.1187	D-bifunctional protein deficiency (MIM#261515)	Molecular
49	SA	0.0313		-0.0421		0.0076	0.0672	Retinitis Pigmentosa (MIM#268000)	Clinical, known X-l + AD genes excluded
50	SA	0.125	0.1629	0.0599	0.1462	0.064	0.1551	3-M syndrome 1 (MIM#273750)	Molecular
51	SA	0.0625	0.1262	0.0296	0.0989	0.0443	0.0944	Junctional epidermolysis bullosa, Herlitz type (MIM#226700)	Molecular
52	SA	0.125	0.1838	0.0723	0.1614	0.0715	0.1524	Retinitis Pigmentosa (MIM#268000)	Clinical, known X-l + AD genes excluded
53	SA	0.125	0.1446	0.0383	0.124	0.0573	0.1177	Hyaline fibromatosis syndrome (MIM#228600)	Molecular
54	SA	0.1875					0.2163	Microcephaly 2, primary, autosomal recessive (MIM#604317)	Molecular
55	SA	0.125	0.1868	0.0486	0.1804	0.0858	0.1479	Retinitis Pigmentosa (MIM#268000)	Clinical, known X-l + AD genes excluded
56	SA	0.0313	0.0879	0.0243	0.0844	0.0231	0.0572	Bardet-Biedl Syndrome 1 (MIM#209900)	Molecular

57	SA	0.25	0.3197	0.0792	0.3038	0.1415	0.2805	Spastic paraplegia 18, autosomal recessive (MIM#611225)	Molecular
58	SA	0.25	0.3846	0.1014	0.3176	0.1557		Bardet-Biedl Syndrome 10 (MIM#209900)	Molecular
59	TUR	0.125	0.1702	0.0637	0.1694	0.0583	0.1248	Citrullinemia (MIM#215700)	Molecular
60	TUR	0.0625	0.0914	0.0061	0.0766	0.0215	0.0782	Muscular dystrophy-dystroglycanopathy (congenital with brain and eye anomalies), type A, 2 (MIM#613150)	Molecular
61	TUR	0.0391	0.1055	0.0255	0.0958	0.0239	0.0682	Ataxia-telangiectasia (MIM#208900)	Molecular
62	TUR	0.2168	0.2396	0.0756	0.2042	0.0962	0.2154	Glycogen storage disease Ib (MIM#232220)	Molecular
63	TUR	0.0625	0.1211	0.0087	0.0972	0.0418	0.108	Muscular dystrophy-dystroglycanopathy (congenital with brain and eye anomalies), type A, 3 (MIM#253280)	Molecular
64	TUR	0.0313	0.0788	-0.0094	0.0464	0.0161	0.0585	Familial Mediterranean fever, AR (MIM#249100)	Molecular
65	MOR	0.0625	0.1219	0.0452			0.0737	3-Methylcrotonyl-CoA carboxylase deficiency (no subtyping)	Biochemical
66	MOR	0.0313	0.091	0.0176			0.0424	Joubert syndrome (no subtyping)	Clinical
67	MOR	0.125	0.2201	0.0873			0.1604	Lipoprotein lipase deficiency (MIM#238600)	Molecular
68	JOR	0,125	0.1646	0.0587			0.1216	Metachromatic leukodystrophy (MIM#250100)	Clinical
69	JOR	0,125	0.0669	-0.0321			0.0581	Methylmalonic aciduria (no subtyping)	Biochemical

70	PAK	0.125	0.1597	0.0476			0.106	Lamellar ichthyosis (MIM#242300)	Molecular
71	NLD	0.0625	0.1753	0.0676			0.1019	Lamellar ichthyosis (MIM#242300)	Molecular
72	AFG	0.1563	0.2057	0.0767			0.1681	Usher syndrome, type 1B (MIM#276900)	Molecular
73	IRN	0.0313	0.0767	0.0012			0.0431	Polycystic kidney disease, infantile, type 1 (MIM#263200)	Molecular

POP= population, Rped = kinship coefficient based on pedigree, PLINK (all), King robust (all), PLINK (by pop.), King homo (by pop.), IBDelphi = R measured by different estimators, AR = autosomal recessive, MIM= Mendelian inheritance in man (www.omim.org).TUN= Tunisia, SAU= Saudi Arabia, TUR= Turkey, MOR= Morocco, JOR= Jordan, PAK= Pakistan, NLD= the Netherlands, AFG= Afghanistan, IRN= Iran

Table S1B. Specification of control couples (n=78)

CONTROL	POP		Rped	PLINK (all)	King robust (all)	PLINK (by pop.)	King homo (by pop.)	IBDelphi
1	TUN	3	0.125	0.1859	0.0715	0.1477	0.0717	0.1551
2	TUN	5	0.0313	0.0596	-0.0033		0.0062	0.0398
3	TUN	3	0.125	0.1407	0.0527	0.1232	0.0547	0.1112
4	TUN	4	0.0625	0.0955	-0.0445		0.0019	0.1254
5	TUN	5	0.125	0.1617	0.0633	0.1163	0.0521	0.1377
6	TUN	5	0.125	0.1094	0.0325	0.0487	0.0229	0.0766
7	TUN	3	0.125	0.1666	0.0679	0.1472	0.0633	0.118

8	TUN	4	0.0313	0.1144	0.0542	0.1083	-0.0058	0.0538
9	TUN	3	0.125	0.1907	0.0405	0.149	0.0736	0.2003
10	TUN	5	0.125	0.2096	0.078	0.1809	0.0894	0.1825
11	TUN	3	0.0625	0.122	0.0526	0.0664	0.0122	0.1043
12	TUN	5	0.0625	0.1472	0.0004	0.1556	0.059	0.1677
13	TUN	5	0.0781	0.0977	0.023	0.0661	0.0291	0.058
14	TUN	4	0.125	0.1541	0.0457	0.1354	0.0666	0.1375
15	TUN	4	0.125	0.1602	0.0611	0.1415	0.0696	0.1245
16	TUN	3	0.125	0.2001	0.0818	0.1667	0.0823	0.1601
17	TUN	5	0.125	0.1504	0.0457	0.142	0.0699	0.1209
18	TUN	4	0.125	0.1897	0.0798	0.1738	0.0794	0.1625
19	TUN	3	0.125	0.1914	0.0668	0.1579	0.0779	0.141
20	TUN	3	0.125	0.1667	0.047	0.1301	0.0639	0.1392
21	TUN	3	0.125	0.1967	0.0747	0.1818	0.0898	0.1687
22	TUN	4	0.125	0.1651	0.0568	0.1419	0.07	0.1415
23	TUN	4	0.0625	0.0887	0.0188	0.0687	0.0331	0.0491
24	TUN	3	0.125	0.1631	0.0582	0.123	0.0604	0.142
25	TUN	4	0.25	0.2644	0.1114	0.2545	0.1264	0.2145
26	TUN	4	0.125	0.1635	0.0612	0.1297	0.0637	0.1188

27	TUN	4	0.125	0.1163	0.0317	0.0985	0.0481	0.0763
28	TUN	5	0.125	0.1715	0.0678	0.1449	0.0704	0.1373
29	TUN	5	0.0625	0.1274	0.0151	0.0937	0.0431	0.1254
30	TUN	4	0.0313	0.0901	0.0108	0.0609	0.0293	0.0475
31	TUN	3	0.0625	0.1489	0.0351	0.1071	0.0525	0.121
32	TUN	4	0.125	0.1661	0.0568	0.1323	0.065	0.1378
33	TUN	3	0.0625	0.0944	0.0223	0.0544	0.0258	0.0583
34	TUN	7	0.0625	0.121	0.0457	0.1049	0.0348	0.0753
35	TUN	3	0.0625	0.0633	0.0025	0.0422	0.0125	0.0216
36	TUN	3	0.125	0.21	0.0872	0.1845	0.0894	0.1809
37	TUN	3	0.125	0.1498	0.0567	0.1204	0.0591	0.114
38	TUN	3	0.125	0.2307	0.0852	0.2051	0.1015	0.191
39	TUN	3	0.0313	0.0927	0.0278	0.086	0.0227	0.0486
40	TUN	3	0.0195	0.0915	0.0134	0.0596	0.0286	0.0601
41	TUN	3	0.0469	0.0938	-0.0064	0.0938	0.031	0.0806
42	TUN	4	0.125	0.1834	0.0447	0.1592	0.0786	0.1634
43	TUN	4	0.1563	0.2298	0.0704	0.215	0.1065	0.2089
44	TUN	4	0.125	0.2156	0.0672	0.1976	0.0978	0.1917
45	TUN	3	0.0156	0.1715	0.0483	0.1361	0.0669	0.1376

46	TUN	3	0.125	0.1996	0.0832	0.1673	0.0827	0.1442
47	TUN	5	0.125	0.0838	-0.0071	0.0922	0.0264	0.0591
48	TUN	4	0.125	0.1757	0.065	0.1552	0.0765	0.1301
49	TUN	4	0.125	0.1696	0.0462	0.1472	0.0726	0.1456
50	SA	4	0.125	0.2164	0.0802	0.1679	0.0794	0.1785
51	SA	7	0.125	0.1853	0.0582	0.1516	0.0714	0.1451
52	SA	3	0.0313	0.0886	-0.0192	0.0729	0.0204	0.115
53	SA	8	0.125	0.1914	0.0821	0.182	0.0821	0.1626
54	SA	3	0.0625	0.1191	0.0288	0.1246	0.0504	0.0801
55	SA	6	0.125	0.2133	0.0894	0.1919	0.0867	0.1789
56	SA	4	0.125	0.2068	0.064	0.1795	0.0854	0.1834
57	SA	6	0.125	0.172	0.0305	0.1358	0.0631	0.1665
58	SA	11	0.125	0.2107	0.0636	0.1917	0.0916	0.1844
59	SA	5	0.25	0.291	0.0871	0.2404	0.1093	0.2337
60	SA	4	0.125		0.0531	0.1861	0.0888	0.19
61	SA	8	0.25	0.32	0.1166	0.3061	0.1492	0.2847
62	TUR	5	0.125	0.1746	0.0374	0.1476	0.0675	0.1501
63	TUR	3	0.125	0.1223	0.0273	0.1095	0.0341	0.0743
64	TUR	3	0.0313	0.0811	0.0103	0.0867	0.0164	0.0243

65	TUR	4	0.125	0.1677	0.0487	0.1468	0.0587	0.1309
66	TUR	3	0.1563	0.1463	0.0395	0.1187	0.0528	0.1164
67	TUR	3	0.1563	0.2063	0.0646	0.1759	0.0806	0.1678
68	TUR	5	0.0313	0.2189	0.0763	0.2035	0.0784	0.1915
69	TUR	3	0.125	0.2031	0.0772	0.1745	0.0724	0.1479
70	TUR	3	0.0313	0.109	0.0265	0.0823	0.0262	0.0573
71	TUR	3	0.1563	0.2187	0.0651	0.188	0.0879	0.1957
72	MOR	3	0.125	0.1999	0.0628			0.1585
73	JOR	9	0.125	0.1881	0.068			0.1575
74	JOR	3	0.0313	0.113	0.0306			0.0806
75	JOR	8	0.125	0.1184	0.0146			0.1044
76	PAK	3	0.0625	0.2236	0.0866	0.2084	0.1013	0.1629
77	NLD	5	0.125	0.1753	0.0551			0.1143
78	IRQ	5	0.1875	0.293	0.1184			0.2569

POP= population, Rped = kinship coefficient based on pedigree, PLINK (all), King robust (all), PLINK (by pop.), King homo (by pop.), IBDelphi = R measured by different estimators, TUN= Tunisia, SAU= Saudi Arabia, TUR= Turkey, MOR= Morocco, JOR= Jordan, PAK= Pakistan, NLD= the Netherlands, IRQ= Iraq.
